# Correlation of pathological examination with indocyanine green (ICG) intensity gradients: a prospective study in patients with liver tumor

**DOI:** 10.1007/s00464-024-10840-9

**Published:** 2024-04-30

**Authors:** Wong Hoi She, Miu Yee Chan, Simon Hing Yin Tsang, Wing Chiu Dai, Albert Chi Yan Chan, Chung Mau Lo, Tan To Cheung

**Affiliations:** Department of Surgery, School of Clinical Medicine, The University of Hong Kong, 102 Pok Fu Lam Road, Hong Kong, China

**Keywords:** ICG, Intensity, Liver resection, Resection margin

## Abstract

**Background:**

Intraoperative indocyanine green (ICG) fluorescence imaging has been shown to be a new and innovative way to illustrate the optimal resection margin in hepatectomy for hepatocellular carcinoma. This study investigated its accuracy in resection margin determination by looking into the correlation of ICG intensity gradients with pathological examination results of resected specimens.

**Methods:**

This was a prospective, single-center, non-randomized controlled study. Patients who had liver tumors indicating liver resection were recruited. The hypothesis was that the use of intraoperative near-infrared/ICG fluorescence imaging would be a promising guiding tool for removing hepatocellular carcinoma with a better resection margin. Patients were given ICG (0.25 mg/kg) 1 day before operation. Resected specimens were inspected under a fluorescent imaging system. Biopsies were taken from tumors and normal tissue. Color signals obtained from ICG fluorescence imaging were compared with biopsies for analysis.

**Results:**

Twenty-two patients were recruited for study. The median size of their tumors was 2.25 cm. One patient had resection margin involvement. Under ICG fluorescence, the tumors typically lighted up as yellow color, wrapped by a zone of green color. Tumors of 17 patients (77.3%) displayed yellow color and were confirmed malignancy, while tumors of 12 patients (54.5%) displayed green color and were confirmed malignancy. Receiver operating characteristic curve was used to measure the sensitivity and specificity of the green color to look for a clear resection margin. The area under the curve was 85.3% (*p* = 0.019, 95% confidence interval 0.696–1.000), with a sensitivity of 0.706 and specificity of 1.000.

**Conclusion:**

The use of ICG fluorescence can be helpful in determining resection margins. Resection of tumor should include complete resection of the green zone shown in the fluorescence image.

Surgical resection is considered the mainstay treatment option for primary liver cancers, including hepatocellular carcinoma (HCC) and intrahepatic cholangiocarcinoma. The primary goal of surgical resection is to achieve a complete R0 resection, which means removing the tumor with clear margins [1, 2]. Similarly, in the treatment of liver metastases, such as colorectal liver metastasis and neuroendocrine tumors, metastasectomy with R0 resection is also sought to improve survival [3].

Currently, liver surgeons primarily rely on intraoperative ultrasound and their tactic sensation to locate the tumors during surgery. They use these methods to palpate the liver and identify the area for transection. However, it is important to note that marking the transection area does not provide information for achieving an R0 resection, which refers to complete tumor removal with clear margins.

Indocyanine green (ICG) has various applications in surgery. When ICG is bound to serum albumin, it can be visualized up to 5–10 mm through body tissue when exposed to near-infrared (NIR) light fluorescence. ICG is exclusively excreted by the liver into the bile. Therefore, in cases of primary liver cancer, the tumor does not excrete ICG and appears as a whole with fluorescence. However, in liver metastasis, the dedifferentiated hepatocytes surrounding the metastasis take up the ICG, resulting in a rim enhancement of fluorescence [4, 5]. This characteristic allows for visualization of the liver, liver tumors [6], biliary tract [7], and vascular perfusion [8]. It also allows for testing of liver function [9] and detection of sentinel lymph nodes [10].

The importance of resection margin in liver tumor resection has been well recorded in previous studies [1–3]. ICG fluorescence imaging has been shown to be a new and innovative way to illustrate the optimal resection margin in hepatectomy for HCC. It can help guide the transection of the liver parenchyma during operation and reduce the risk of early recurrence. In addition, ICG is safe and the risk of complication of ICG use is low [11]. In this study, we investigated whether intraoperative ICG fluorescence imaging helps in the achievement of a clear resection margin, thereby reducing tumor recurrence.

## Methods

The study protocol was approved by the institutional review board, and written informed consent was obtained from all participating patients.

This prospective, single-center, non-randomized controlled study was conducted at Department of Surgery, The University of Hong Kong, from September 2021 to January 2023. Patients who had liver tumors indicating liver resection were recruited for the study.

Patients were given ICG (0.25 mg/kg) 1 day before liver resection. A fluorescent imaging system (NIR/ICG system FDA 510 (k), KARL STORZ, Germany) was used to examine resected specimens. In each case, two tissue biopsies were taken, one from the tumor and another from normal tissue. The color signal obtained from the ICG fluorescence imaging was compared with the biopsy samples for further analysis.

Descriptive data regarding tumor characteristics, operative details, postoperative outcomes, oncological outcomes and survival were recorded and listed as part of the study analysis.

### Hypothesis

The use of intraoperative NIR/ICG fluorescence imaging would be a promising guiding tool for removing HCC with a better resection margin.

### Research plan and methodology

This study was initiated by the investigators and conducted at Department of Surgery, The University of Hong Kong. Recruitment of patients was done by the principal investigator and co-investigators. Patients with newly diagnosed or recurrent liver tumors were recruited according to the following inclusion and exclusion criteria.

### Inclusion criteria

- Hepatectomy was indicated
- Laparoscopic or open liver resection was considered technically feasible
- Adequate liver reserve
- Absence of extrahepatic metastasis
- Absence of radiological evidence of venous invasion of the major portal vein or hepatic vein branches
- Liver function of Child–Pugh class A or B
- Karnofsky performance status ≥ 70%
- Total serum bilirubin ≤ 3 × upper normal limit
- Platelet count > 60 × 10^9^/L
- Serum creatinine ≤ 2 × upper normal limit

### Exclusion criteria

- Presence of extrahepatic metastasis
- Unsuitability for general anesthesia
- Refusal to consent
- Presence of previously documented allergy to ICG

Resectability was determined based on the anatomical location of the tumor in relation to major hepatic vasculatures, regardless of tumor size. The Couinaud nomenclature was used to define major and minor resections. The criteria for selecting patients for major resection have been previously reported [9]. In cases where there was insufficient liver volume, portal vein embolization or ALPPS was considered. To assess liver anatomy and identify any systemic involvement, computed tomography or magnetic resonance imaging was conducted, sometimes supplemented with dual-tracer positron emission tomography in the later period of the study. Postoperative complications were graded according to the Clavien–Dindo classification [12].

Resections were carried out using the open or laparoscopic approach, with laparoscopy being the preferred method whenever feasible. The specific techniques for open and laparoscopic resections have been detailed in previous descriptions [13]. In cases where tumors involved the bile duct, en bloc resection of the tumor and the bile duct was performed. This was followed by biliary reconstruction using hepaticojejunostomy.

### Administration of ICG

For patients who underwent liver resection more than 2 weeks after the initial ICG retention test, an additional intravenous injection of ICG (0.25 mg/kg) was administered 24 h prior to the operation.

### Fluorescent imaging system

**Fig. 1:**
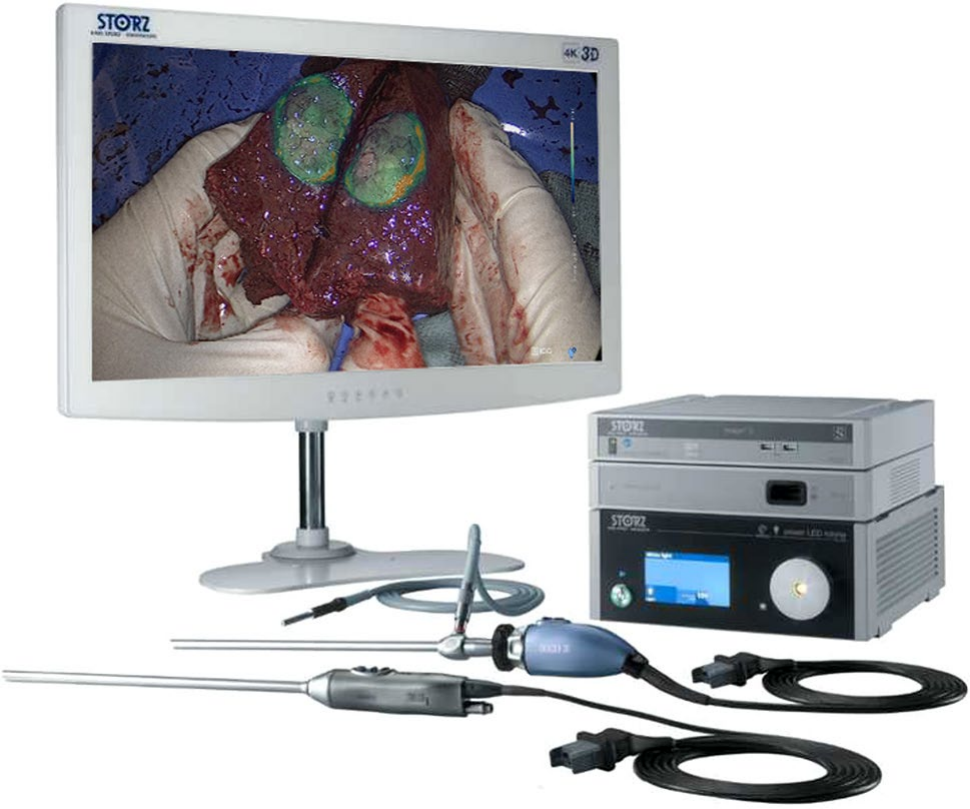
The ICG system used in the study

The endoscopic fluorescent imaging system (NIR/ICG System using the OPAL1® technology with the modular IMAGE1 S™ system, FDA 510(k), KARL STORZ, Germany) includes a 10-mm laparoscope, camera head, xenon light guide cable, video processor/illuminator, and 4 K-3D monitor. The charge-coupled device camera and xenon light source in the system have the ability to filter out light wavelengths below 810 nm and above 800 mm. This allows for optimal visualization of NIR light and has the capability to switch images from full floor to fluorescence images, enabling the detection and visualization of fluorescence during the surgical procedure. (Fig. 1).

### Harvesting tissue samples from resected specimens for histopathological evaluation

**Fig. 2:**
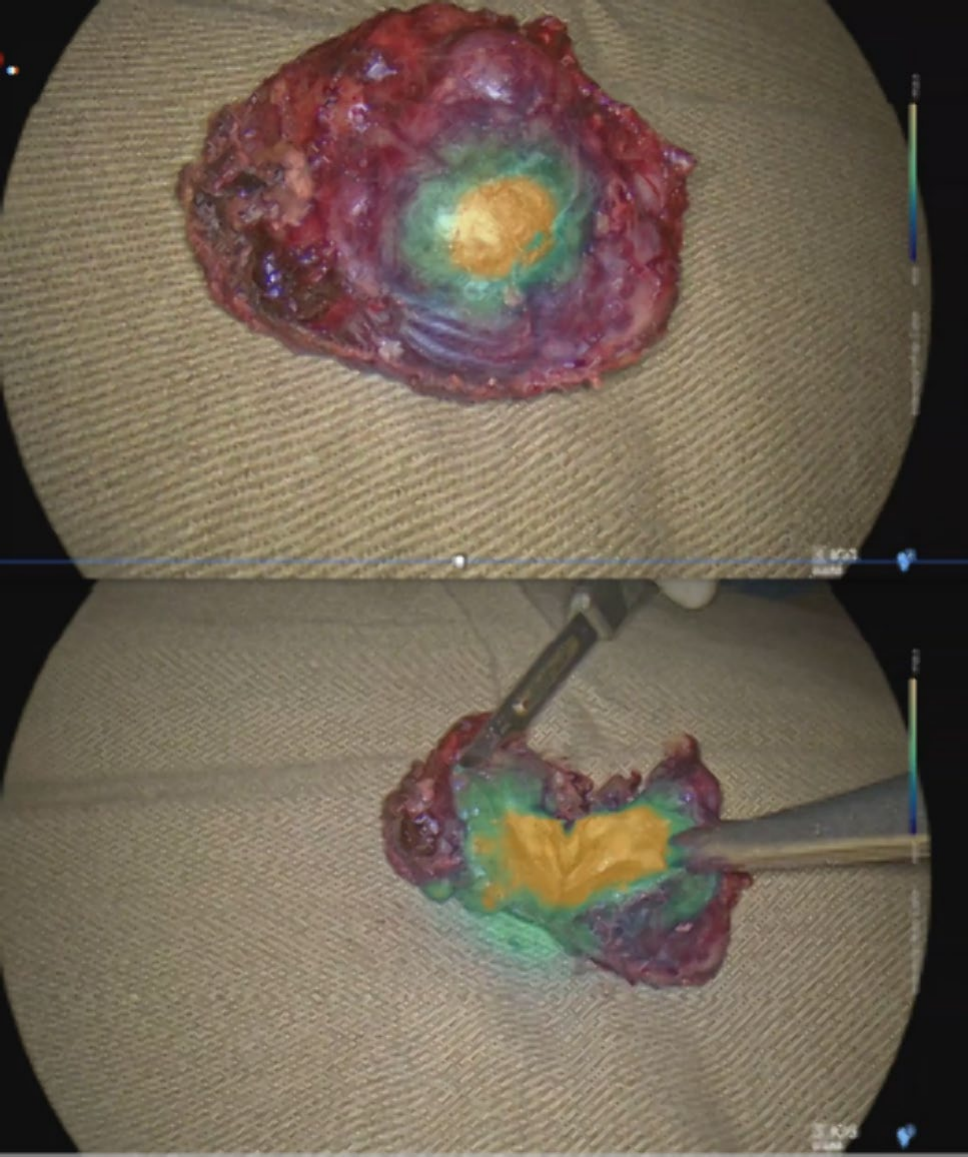
Example of resected tumor under ICG fluorescence (the hepatic specimen incised, confirming resection of the HCC)

Two tissue biopsies, one from the tumor lesion and another form the normal tissue adjacent to the tumor, were collected from the resected specimen and sent for histological assessment. (Fig. 2).

The pathological tissue samples, which were obtained from paraffin-embedded liver sections, were examined by at least two experienced pathologists who were blinded to the results of the radiological examinations. The presence of venous permeation was recorded if tumor emboli were identified within the liver’s venous system, including the portal vein, its lobar and segmental branches, the hepatic vein, capsular vein, and inferior vena cava. The tumor was histologically graded according to the Edmondson criteria.

### Follow-up

All patients were subjected to regular follow-up examinations, with monthly follow-ups conducted in the first year and quarterly follow-ups thereafter if no recurrence was detected. The first computed tomography or magnetic resonance imaging was performed approximately 1 month after the resection and subsequent scans were repeated every three to 4 months during the first year and every 6 months in the following years. The diagnosis of recurrence was primarily based on the identification of typical imaging findings on computed tomography or magnetic resonance imaging. If necessary, percutaneous biopsy was performed to confirm the recurrence. This approach allowed for timely detection and management of recurrent tumors.

### Statistical analysis

Descriptive data of tumor characteristics, operative details, postoperative and oncological outcomes, and survival were recorded. Continuous variables were expressed as median with interquartile range. Pearson’s chi-squared test or Fisher’s exact test, where appropriate, was used to compare categorical variables. Receiver operating characteristic (ROC) curves were constructed to evaluate the adequacy of resection margins by visualization of the tumors using ICG fluorescence. The area under the curve was calculated to determine the sensitivity and specificity of ICG fluorescence in assessing the resection margin. The computer software SPSS, version 24.0, was used for statistical analyses. A p-value of less than 0.05 was considered statistically significant.

### Consent

Prior to operation, every patient was given full explanation of the trial before they gave their voluntary written consent.

### Conflicts of interests

None of the investigators had any financial interests in the materials or equipment used in the trial. The trial did not receive any financial support from any organization that had any relationship with the materials or equipment used in the trial.

## Results

Twenty-two patients (18 men and 4 women) who underwent liver resection were studied. Their median age was 63 years. Most of them (81.8%) carried hepatitis B virus. All patients had Child–Pugh class A liver function. Most patients (86.4%) underwent minor hepatectomy, and 68.2% of the patients underwent laparoscopic resection. Approximately, one-third of the patients (31.8%) underwent re-resection for recurrent HCC. During the Intraoperative phase, ICG fluorescent imaging in an intensity mode was used for tumor visualization. Complications related to surgery occurred in 3 patients, including 2 patients who had major complications according to the Clavien–Dindo classification. The median length of hospital stay was 4.5 days.

In terms of pathology, 18 patients (81.8%) had HCC, one patient had intrahepatic cholangiocarcinoma, one had focal nodular hyperplasia, one had neuroendocrine tumor, and one had reactive lymphoid hyperplasia. The median number of tumors was one, and the median tumor size was 2.25 cm. One patient (4.5%) had an involved surgical margin, and the median resection margin width was 0.375 cm. Chronic hepatitis was observed in 6 patients (27.3%) and 10 patients (45.5%) had cirrhosis. No macrovascular invasion was detected, but 3 patients (13.6%) had evidence of microvascular invasion. The tumor staging according to the UICC, 8th edition, is provided in Table 1.

**Table 1:** Patients’ characteristics

Patients’ characteristics	Study cohort (*n* = 22)
Age (years)	63.0 (35–80)
Male:Female	18:4
Hepatitis B surface antigen	
No	4 (18.2%)
Yes	18 (81.8%)
Child–Pugh class A	22 (100%)
Operative details	
Blood loss (L)	0.3 (0.01–1.65)
Blood replacement (L)	0.0 (0.0–0.0)
Blood transfusion	0 (0%)
Operation time (min)	153.5 (60–400)
Resection extent	
Major resection	3 (13.6%)
Minor resection	19 (86.4%)
Surgical mode	
Open	7 (31.8%)
Laparoscopic	15 (68.2%)
Pringle maneuver	
No	20 (90.9%)
Yes	2 (9.1%)
Re-resection	7 (31.8%)
Rubina ICG fluorescence with tumor signal	
Yellow color	17 (77.3%)
Green Color	12 (54.5%)
Normal parenchymal color	0 (0%)
Overall complication	3 (13.6%)
Complication of grade IIIA or above	
IIIA	1 (4.5%)
IIIB	1 (4.5%)
Hospital stay (d)	4.5 (1–13)
Pathological details	
No. of tumor nodule	1 (1–3)
Tumor size (cm)	2.25 (1.2–11.5)
Narrowest resection margin (cm)	0.375 (0.0–2.5)
Margin involvement	1 (4.5%)
Liver status	
Non-cirrhotic	6 (27.3%)
Chronic hepatitis	13 (59.1%)
Cirrhotic	3 (13.6%)
No macrovascular invasion	22 (100%)
Microvascular invasion	
Absent	19 (86.4%)
Present	3 (13.6%)
Bilobar disease	1 (4.5%)
UICC8	
IA	3 (13.6%)
IB	3 (13.6%)
II	2 (9.1%)
IIIA	1 (4.5%)
IIIB	3 (13.6%)

Data presented as medium (range) or number of patient (percent) or ratio

**Fig. 3:**
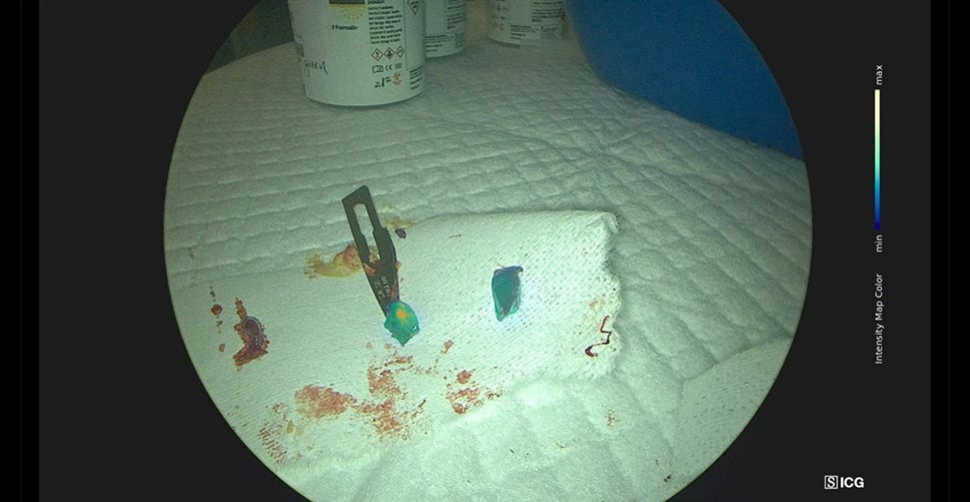
Normal tissue (left), yellow tumor (middle), and green tumor (right) (color figure online)

**Fig. 4:**
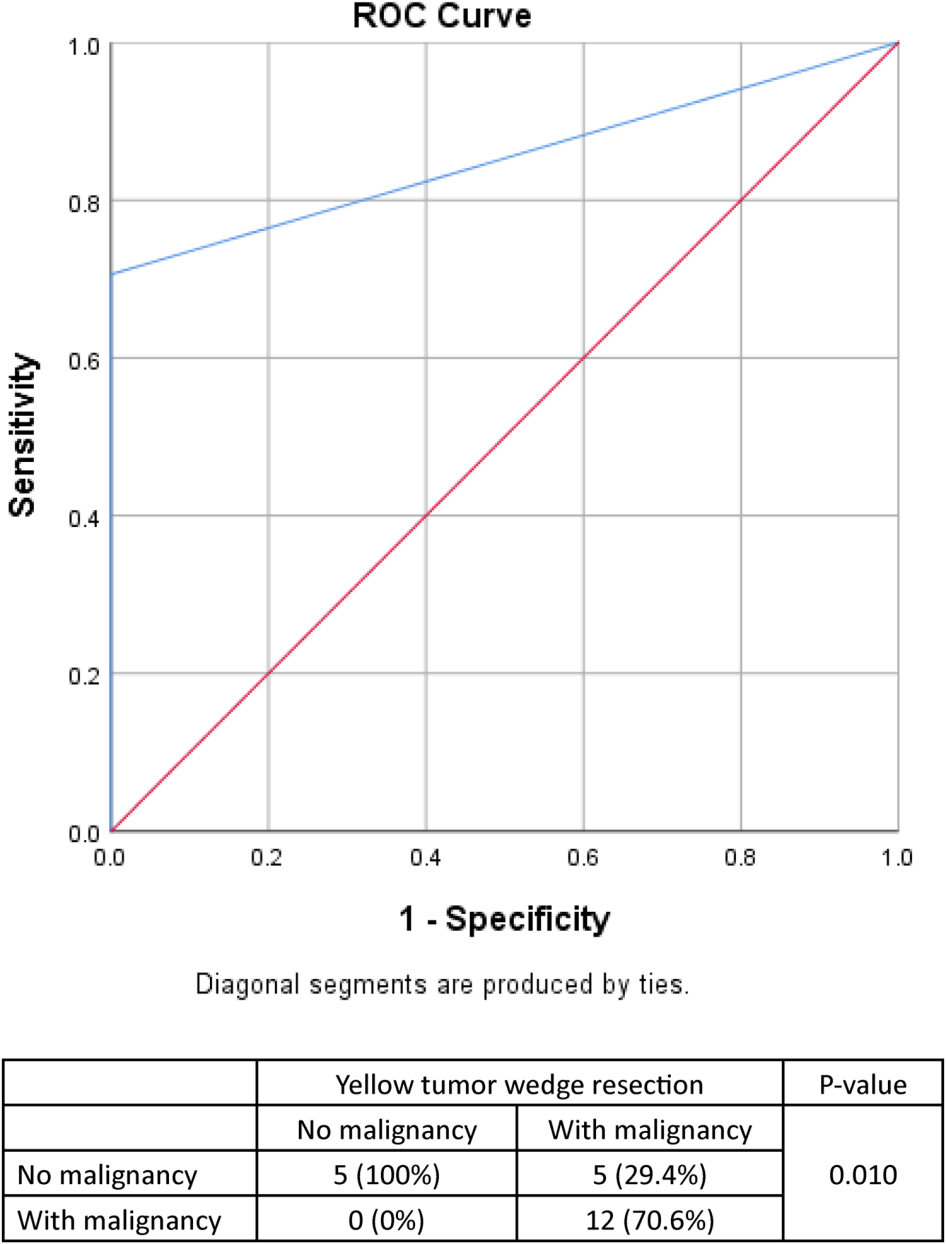
ROC graphical approach for yellow ICG fluorescence in correlation with tumor pathology (the area under the ROC curve was 85.3%, *p* = 0.019, 95% confidence interval 0.696–1.000, with a sensitivity of 0.706 and specificity of 1.000)

Under fluorescent imaging, a tumor typically appears as yellow color and is surrounded by a zone of green color (Fig. 3). In this study, 17 tumors (77.3%) displayed yellow color, confirming their malignancy. Additionally, 12 tumors (54%) displayed green color, also confirming malignancy. ROC analysis was performed to evaluate the sensitivity and specificity of ICG fluorescence in detection of tumor involvement. The area under the ROC curve was 85.3%, indicating a good discriminatory ability of ICG fluorescence in identifying tumor involvement. The p-value was 0.019 (95% confidence interval 0.696–1.000), with a sensitivity of 0.706 and specificity of 1.000, suggesting a high level of accuracy in the detection of tumor presence using ICG fluorescence. (Fig. 4).

## Discussion

The primary goal of a hepatectomy is to achieve a negative resection margin, which has been demonstrated to be a significant prognostic factor [14]. Lai et al. [1] reported that a resection margin wider than 0.5 cm was considered ideal, particularly for multinodular lesions. On the other hand, some studies reported that a resection margin narrower than 1 cm was associated with a higher rate of tumor recurrence and shorter survival. However, all these findings highlighted the importance of an adequate resection margin in optimization of outcomes in patients [2, 15, 16].

The use of ICG in assessing resection margin intraoperatively had been reported [17]. However, the correlation between the intensity map image obtained using ICG fluorescence and pathological findings had not been well established. This study demonstrated the benefits of using ICG fluorescence in surgical procedures. During surgery, tumors were visualized under fluorescence imaging and appeared as yellow in color. The yellow color represented the central part of the tumor. Surrounding the yellow zone, a green-colored area was observed under fluorescence imaging. This green zone could represent either a tumor-free area or a positive resection margin. The study found that the correlation between color changes observed in the intensity map of ICG fluorescence and pathological findings was significant. The yellow zone accurately represented the central portion of the tumor, while the green zone surrounding it corresponded to the surrounding tissue, which could potentially be tumor free or indicate a positive resection margin. These findings highlighted the potential of ICG fluorescence in providing real-time visualization and assessment of the resection margin during surgery, aiding in the determination of tumor involvement and the achievement of negative resection margins.

This real-time visualization provided by ICG fluorescence serves as a valuable roadmap during tumor resection. It helps guide the surgeon in determining the transection line, ensuring an adequate resection margin of 1 cm. It is important to note that ICG has a tissue penetration depth of up to 10 mm. Due to the limited tissue penetration depth of ICG, not every ICG-positive spot indicating tumor infiltration can be visualized on the liver surface. However, the presence of ICG staining alerts the surgeon to be cautious about the transection zone within 0 to 10 mm from the resection margin. This caution is necessary because this zone could be too close to the tumor or may contain small satellite nodules. Using ICG fluorescence, the surgeon can identify regions in close proximity to the tumor and areas of potential tumor involvement, which helps with their decision-making during the resection. ICG fluorescence imaging is useful in ensuring an adequate resection margin and reducing the risk of leaving behind tumor cells or overlooking satellite nodules that could affect the patient’s prognosis.

Currently, there is no effective means of determining the resection margin intraoperatively. Tactile sensation with gentle palpation can assist in localizing the tumor and guiding the transection. Intraoperative ultrasound can improve the precision of the transection line by marking the site of transection away from the tumor, thereby enhancing the surgical margin. With the assistance of ICG fluorescence, the surgeon can adjust the transection plane to ensure an appropriate surgical margin. ICG fluorescence enables visualization of the tumor and the surrounding tissue, allowing for real-time assessment of tumor involvement. This additional information helps the surgeon make informed decisions during the procedure to achieve an adequate surgical margin.

NIR light image-guided surgery can directly guide the surgeon to the target area if the tumor is located on the liver surface or is within 10 mm in depth. For tumors located deeper in the liver, ICG visualization can inform the surgeon if the transection is approaching too close to the surgical margin. In case of doubt, a piece of liver tissue from the transection zone can be sent for frozen section to confirm tumor clearance. Persistent display of fluorescence on the transection surface of the remnant liver may suggest the presence of residual tumor tissue [18], indicating the need for further resection or intervention. By combining NIR light image-guided surgery, ICG visualization, and frozen section analysis, surgeons can enhance their ability to achieve clear resection margins and ensure effective tumor removal during liver surgery.

The intensity map mode of the ICG fluorescence allows for visualization of tumor zones. According to the experience gained in this study, liver resection should encompass these zones to ensure a safe and margin-free resection. The more centrally located yellow zone represents the main bulk of the tumor. The likelihood of obtaining a positive pathological result from a specimen stained yellow is higher. On the other hand, the tumor is surrounded by a zone of green fluorescence. This area can indicate a tumor-free region wrapping around the tumor capsule, resulting in a lower rate of malignancy detection. Including a region of normal tissue surrounding the green-colored tumor is crucial for achieving a safe resection with an adequate surgical margin. By taking into account, the distinct zones visualized through ICG fluorescence, surgeons can improve the precision of the resection, ensuring complete removal of the tumor while maintaining an appropriate margin of healthy tissue. This approach supports a safe and effective surgical procedure.

This study, despite its relatively small sample size, demonstrated the usefulness of ICG fluorescence in assisting liver resection. It was proven beneficial in increasing the likelihood of achieving an R0 resection in all types of tumors requiring hepatectomy, thereby improving patient outcomes. However, the limited depth of ICG fluorescence remains a challenge. In scenarios where deeper lesion resection is necessary, surgeons still rely on tactile sensation and intraoperative ultrasound to mark the initial transection before proceeding to use ICG fluorescence as an adjunct for further resection. While ICG fluorescence provides valuable assistance in liver resection, its limitations for deeper lesions necessitate the integration of other techniques, such as palpation and ultrasound, to ensure accurate identification and removal of the tumor. By combining these methods, surgeons can optimize surgical outcomes and enhance the effectiveness of liver resection.

## Conclusion

The use of ICG fluorescence can be helpful in determining resection margins. Resection of tumor should include complete resection of the green zone shown in the fluorescence image.
